# Studying Alzheimer disease, Parkinson disease, and amyotrophic lateral sclerosis with 7-T magnetic resonance

**DOI:** 10.1186/s41747-021-00221-5

**Published:** 2021-08-26

**Authors:** Emrah Düzel, Mauro Costagli, Graziella Donatelli, Oliver Speck, Mirco Cosottini

**Affiliations:** 1 Otto-von-Guericke University Magdeburg, Magdeburg, Germany; 2 German Center for Neurodegenerative Diseases (DZNE), Magdeburg, Germany; 3 University College London, London, UK; 4 IRCCS Stella Maris, Pisa, Italy; 5 University of Genoa, Genova, Italy; 6 Fondazione Imago 7, Pisa, Italy; 7 Azienda Ospedaliero Universitaria Pisana, Pisa, Italy; 8 University of Pisa, Pisa, Italy

**Keywords:** Alzheimer disease, Amyotrophic lateral sclerosis, Magnetic resonance imaging, Neurodegenerative diseases, Parkinson disease

## Abstract

Ultra-high-field (UHF) magnetic resonance (MR) scanners, that is, equipment operating at static magnetic field of 7 tesla (7 T) and above, enable the acquisition of data with greatly improved signal-to-noise ratio with respect to conventional MR systems (*e.g.,* scanners operating at 1.5 T and 3 T). The change in tissue relaxation times at UHF offers the opportunity to improve tissue contrast and depict features that were previously inaccessible. These potential advantages come, however, at a cost: in the majority of UHF-MR clinical protocols, potential drawbacks may include signal inhomogeneity, geometrical distortions, artifacts introduced by patient respiration, cardiac cycle, and motion. This article reviews the 7 T MR literature reporting the recent studies on the most widespread neurodegenerative diseases: Alzheimer’s disease, Parkinson’s disease, and amyotrophic lateral sclerosis.

## Key points

- Ultra-high-field MRI enables improved signal-to-noise ratio, resolution and tissue contrast.
- In Alzheimer disease, 7-T MRI enables high-resolution assessment of neurodegenerative processes affecting hippocampal structures as well as vascular lesions and vascular reserve.
- 7 T imaging of substantia nigra has outstanding accuracy in identifying Parkinson disease patients.
- In Amyotrophic lateral sclerosis, 7-T MRI reveals motor neuron impairment signs in cerebral cortex.

## Introduction

Magnetic resonance (MR) is used in medicine since nearly four decades ago. While its ability to represent soft tissues *in vivo* non-invasively has had a crucial impact on clinical diagnosis since its early days, the steady quest for improved data quality and signal-to-noise ratio (SNR) has led to the recent use, in clinical studies, of MR scanners operating at ultra-high field (UHF) of 7 T and beyond.

In imaging (MRI) applications, the main advantage of higher SNR is the increased sensitivity to signal changes related to tissue composition and physiological parameters [1]. The higher SNR, which increases linearly with the static magnetic field strength, enables also to achieve improved spatial resolution. Another important feature in UHF MR is the change in tissue relaxation times: in particular, the combination of higher SNR and shorter T2* at UHF has been exploited to obtain images with unprecedented anatomical detail in susceptibility-weighted imaging (SWI) and quantitative susceptibility mapping (QSM) [2]. In functional MRI (fMRI), these two features have enabled researchers to obtain activation maps based on blood oxygenation level dependent (BOLD) contrast with sub-millimetric resolution [3, 4]. Such an increased sensitivity to magnetic susceptibility is, however, also a source of undesired effects, primarily signal loss at tissue interfaces [5], vulnerability to artifacts introduced by patient respiration and cardiac cycle [6], motion [7], and geometrical distortions [8]. Techniques to mitigate these effects have not yet been implemented in the majority of clinical scenarios.

Another challenge in UHF MRI is posed by the shortening of the resonance wavelength at UHF, which can cause signal inhomogeneity at spatial scales of the size of the human head. This problem has been solved with parallel transmission [9] at a number of UHF MR sites; however, this technology is not yet available in the most part of clinical contexts.

In MR spectroscopy (MRS), besides the increase in SNR, one main advantage of operating at UHF is the increase in spectral resolution [10]. Further, as the resonance frequency increases linearly with the static magnetic field, operating at UHF also facilitates MRI and MRS of other nuclei, such as ^23^Na and ^31^P, whose abundance (hence, MR signal) and resonance frequencies are far lower than those of the ^1^H proton. In this context of intertwined potential advantages and challenges, this article aims to provide an overview of recent results and future perspective of UHF MR in clinical studies addressing three major neurodegenerative diseases, namely Alzheimer’s diseases, Parkinson’s disease, and amyotrophic lateral sclerosis.

## Alzheimer disease

Alzheimer disease (AD) is characterized by long preclinical and prodromal stages with progressive molecular pathology, neurodegeneration and cognitive impairment. The ATN (amyloid, *T*au, *n*eurodegeneration) [11] research framework considers ß-Amyloid, Tau-pathology, and neurodegeneration (neuronal or synaptic loss, atrophy) as the hallmarks of clinical diagnosis and individual staging for the purpose of clinical trials. While the levels of ß-amyloid and tau-pathology can be determined using cerebro-spinal fluid (CSF), plasma, and molecular imaging methods [12], determining the degree of neurodegeneration (as defined in the ATN framework) remains challenging and 7-T magnetic resonance imaging could provide a substantial advantage over MRI at 1.5 or 3 T. In addition to assessing neurodegeneration, 7-T imaging provides innovative readouts for vascular pathology occurring either as a consequence of AD or as a comorbidity, for dysfunction of macro- and mesoscale neural networks and for molecular pathology.

### Assessment of neurodegeneration in AD

**Fig. 1:**
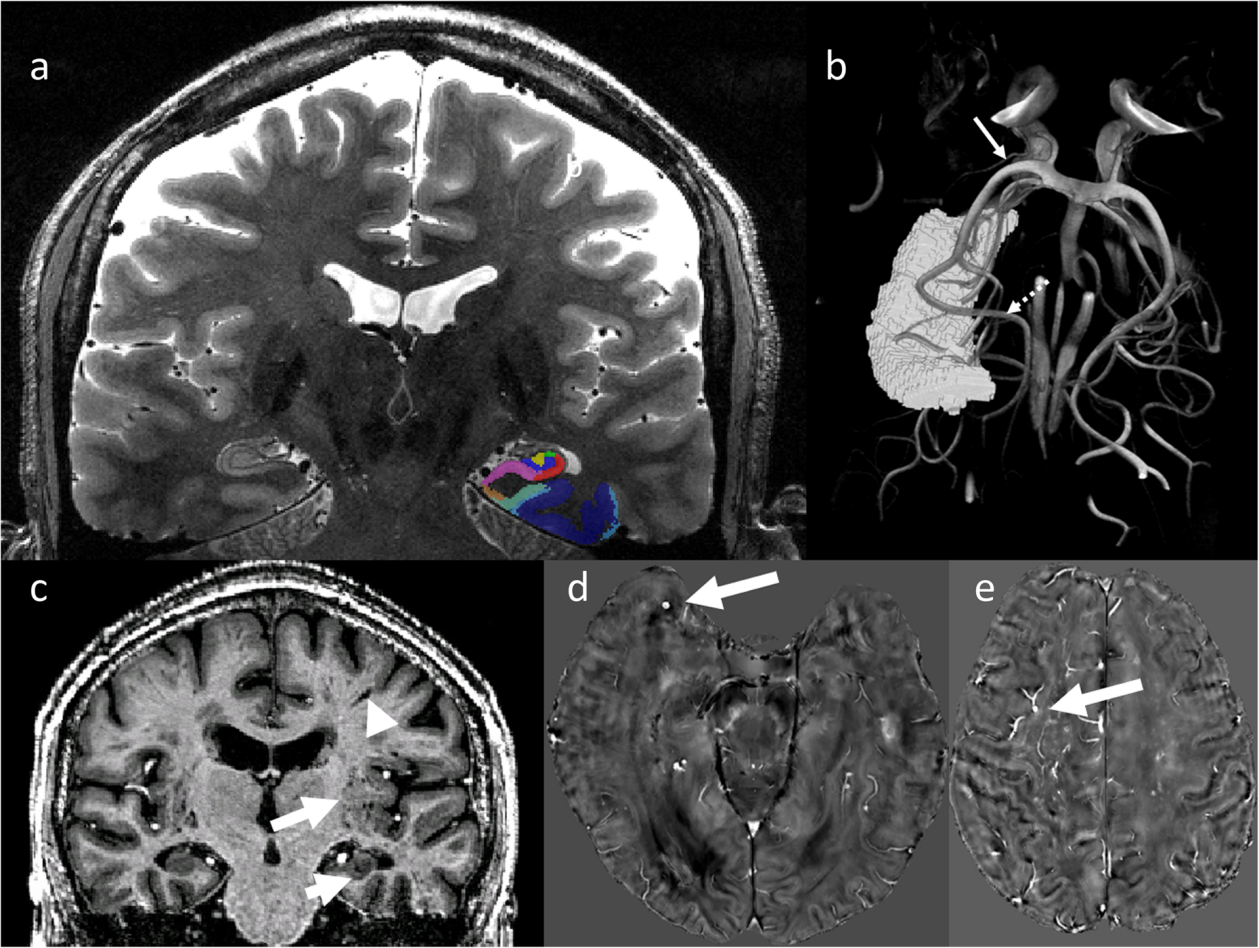
Structural and vascular hippocampal and medial temporal lobe imaging at 7T in an older adult. **a** T2-weighted coronal 7-T MRI scan through the body of the hippocampus, immediately distal to the hippocampal head. Color legend of segmented regions: entorhinal cortex brown, Brodmann area 35 (transentorhinal) teal, Brodmann area 36 dark blue, subiculum mauve, CA1 red, CA2 green, CA3 yellow, dentate gyrus blue. **b** High-resolution time of flight imaging of hippocampal vascularization allowing to identify supplying vessels from the anterior choroidal artery (solid arrow) and the posterior cerebral artery (dashed arrow). **c** A structural T1-weighted coronal 7-T MRI in a patient with mild cognitive impairment and markedly enlarged perivascular spaces, particularly in the insular regions (long arrow) but also in the hippocampus (middle arrow). The scan also shows linear perivascular spaces alongside vessels (short arrow). **d** A microbleed in the anterior temporal lobe (white arrow) in a patient with cerebral amyloid angiopathy imaged with QSM at 7T. **e** A microbleed with a venous connection, as visualized with a QSM based venography

Measures of cortical and subcortical grey matter volume or thickness with MRI and their progression over time are likely to be the most direct measures of local neurodegeneration that are currently available. Structural MRI with visual inspection at 1.5 and 3 T has been at the heart of diagnostic radiology in dementia for two decades, while volumetric analysis from structural imaging has been the principal imaging marker of major cohort studies and trials of disease modifying therapies in symptomatic sporadic dementia with approval by the European Medicines Agency [13, 14] and pre-symptomatic genetic dementias [15]. Structural sequences have proven sufficiently robust to site and even manufacturer effects, to allow large scale multi-center collaborative studies. Following this widespread use, there is extensive modelling and empirical evidence for the power of T1- and T2-weighted imaging to detect rates of change and the effect of treatment, for a given cohort size, study duration, and drug effect [16]. 3 T structural MRI has become an industry standard surrogate marker for drug trials in Alzheimer’s disease. However, 1.5 T and 3 T structural imaging is fundamentally limited by its coarse resolution (typically about 1 mm; macro-scale) and contrast-to-noise ratio (SNR), which prevents accurate quantification of volumetric change within short-intervals or pre-symptomatic change, and accurate delineation of medial temporal sub-region and hippocampal subfield changes that characterize early stage neuropathology. This has been highlighted recently outlining the inadequacy of the common use of 3T-based 1 mm isotropic MRI to perform hippocampal subfield segmentations [17] using automated or manual methods. Given that cortical thickness is around 2–3 mm, the limited sensitivity of current structural imaging to detect cortical atrophy is apparent. Efforts are underway to harness the superior resolution of 7 T for AD research. Examples for high-resolution structural imaging scans that have been successfully implemented in multi-center studies and across vendors, *e.g.,* in the setting of the EUFIND (European ultra-high-field imaging network for neurodegenerative diseases) consortium, include a T1-weighted magnetization-prepared rapid gradient echo sequence for whole-brain anatomy (3D MPRAGE, 0.65-mm isotropic resolution), and a T2-weighted acquisition centered on the medial temporal lobe (2D Turbo-Spin Echo, 0.4 × 0.4 × 1.0 mm resolution, orthogonal to the hippocampus’ longest axis) [18]. As illustrated in recently developed 7-T segmentation protocols for the medial temporal lobe (MTL) [19], certain key landmarks that are difficult to identify at 3T, such as the endfolial pathway distinguishing dentate gyrus from hippocampal subfield CA3, can be identified reliably in 7-T scans using these types of resolution (Fig. 1). Other structures for which morphometric quantification of volume or thickness is difficult at 3 T include subregions of the entorhinal cortex [20, 21] and the transentorhinal cortex, which are affected early on in the pathological cascade of AD [12, 22–24]. These initial efforts revealed two acquisition problems, namely excessive head motion and signal loss in the inferior temporal lobe on T2-weighted scans. Future solutions could include prospective motion correction [25] and utilization of parallel transmission to homogenize the transmit field [9] and these solutions are indeed likely to be commercially available in foreseeable time.

While the benefits of 7 T for assessing cortical and subcortical morphometry at the macro-scale are ready-to-use for studies in AD, the high-resolution of 7 T could also provide new, more mechanistic insights into how neurodegeneration progresses across brain regions by allowing to quantify structural integrity in different cortical layers by way of its submillimeter (meso-scale) resolution. Such quantification of laminar thickness and its changes in disease and with age and disease pathology [26, 27] can reveal microstructural insights into the causal cascade of neurodegeneration because of the layer-specific organization of feedforward and feedback connectivity [28].

Another emerging area of advanced structural imaging at 7 T that is relevant for AD is imaging of the locus coeruleus (LC). The LC is the sole origin of cerebral noradrenergic supply, and one of the first sites of the human brain to develop neurofibrillary tangles in preclinical AD (for a review see [29]). Studies at 3 T have already showed that the LC MRI contrast is reduced in AD in proportion to CSF Aβ levels [30]. Given the small size of the LC and its limited contrast, efforts are made to image its structural and functional integrity at 7 T [29] for instance by developing optimized magnetization transfer (MT)-weighted imaging approaches (*e.g.*, [31]).

### Vascular system and vascular pathology

Vascular pathology is an important risk factor and comorbidity that can modify neurodegeneration and disease progression in AD [11]. While it can be associated with neurodegeneration even in the absence of Alzheimer’s disease pathology [32, 33], it is now clear that there is an interaction between vascular and non-vascular pathology in AD. Indeed, vascular pathology has been proposed as an important driver of neurodegeneration for amyloid-positive but tau-negative individuals [11]. Besides established markers of small vessel disease (SVD) at 3 T (white matter hyperintensities, lacunes, microbleeds [34, 35], perivascular spaces), ultra-high-resolution imaging offers new possibilities to quantify vascular pathology and vascular reserve. Corresponding ultra-high-resolution sequences have been implemented in multi-center networks such as EUFIND. Measures technically feasible at 7 T include Fluid-Attenuated Inversion Recovery (FLAIR), T2 and T1 to image white matter hyperintensities, microinfarcts (hyperintense on FLAIR and hypointense on T1 MPRAGE) [36, 37], perivascular spaces, diffusion tensor imaging (DTI) to infer information about axonal integrity in the vicinity of white matter hyperintensities, ultra-high-resolution 2D phase-contrast imaging to assess the pulsatility of perforating arteries [37–42], QSM to image microbleeds (spherical hypointensities on the magnitude images of the QSM datasets) [34, 35], venous vessel density, length, tortuosity and branching patterns, and time-of-flight (TOF) angiography to measure small arterial features including the hippocampal small-vessel vascularization patterns [43, 44].

Enlarged perivascular spaces (Fig. 1c), which are enlarged pathways of clear interstitial fluid, while still unclear whether they represent perivenular or periarteriolar phenomena [45], are likely to indicate a failure to clear fluid and waste, including amyloid and tau protein [46–48]. Combinations of FLAIR, T2-weighted, and susceptibility-weighted imaging submillimeter resolution venography and TOF angiography with high resolution at 7 T [49, 50] could provide new insights into pathological progression of vascular dysfunction in AD and the interaction between small vessel disease and AD. For instance, high-resolution 7-T assessments of the progressive build-up of perivascular spaces alongside the temporal progression of amyloid and tau pathology could indicate whether dysfunction in clearance precedes the progression of amyloid and tau pathology or is a consequence of its progression (*i.e.*, more waste products to clear). Furthermore, in combination with structural imaging of cortical neurodegeneration (see above), 7 T could help to assess individually whether progression of neurodegeneration is related primarily to AD pathology (amyloid and tau) or concomitant vascular disease. A quantitative vascular profile including white matter hyperintensity volume, number of microinfacts, number of microbleeds (Fig. 1d), mean length and tortuosity of arteries and veins, mean venous density, number of perforating arteries, mean perforating artery velocity and mean perforating artery pulsatility index, perivascular spaces, and their spatial distribution, could be assessed and related to neurodegeneration and the progression of amyloid and tau pathology. Given the superior resolution of 7 T, it can be expected to assess this prognostic question with higher sensitivity and in a shorter time period than with 3 T, but this needs to be demonstrated in comparative studies. Given the advent of disease modifying treatments targeting amyloid-pathology, such individualized assessments of the cause of neurodegeneration could have important impact for therapeutic decisions in a personalized medicine framework. Finally, a clinically important question to which this type of multimodal imaging could contribute is the differential diagnosis of small vessel disease and cerebral amyloid angiopathy (Fig. 1e).

Recently, it has been shown that 7-T high-resolution TOF-angiography enables to classify individual hippocampal vascularization patterns [43, 44]. Five hippocampal vascularization patterns can be distinguished, according to the origin of the hippocampal arteries. We have shown that the 7T-based *in-vivo* classification yields similar results as former post-mortem studies and that individuals whose hippocampus is supplied by one vessel system (posterior cerebral artery) as opposed to two systems (also anterior choroidal artery) and have cerebral small vessel disease, have poorer cognitive scores [43]. Hence, 7 T may provide a window to individually assess hippocampal vascular reserve and thus opens new perspectives for personalized risk modification and disease management. In this context, the interaction between vascular reserve and progression of Alzheimer’s disease could also be particularly relevant. It can be hypothesized that individuals with lower hippocampal vascular reserve as determined on the basis of hippocampal vascular supply patterns, could suffer steeper cognitive decline (fast progressor) with advancing amyloid and tau pathology.

### Functional imaging

While synapse loss correlates closely with symptoms in Alzheimer’s disease [51] and therefore neurodegeneration is an important target of MRI imaging, it is also well established that amyloid and tau-pathology can impair brain function through synaptotoxicity. Animal studies show that misfolded and hyperphosphorylated tau can impair neuronal function [52]. Mislocation of tau to dendritic spines can cause synaptic dysfunction [53] and there is evidence that pathological tau reduces network activity [54]. This is well compatible with the reduction of a novelty response in the hippocampus and amygdala as recently reported to be independent of hippocampal and amygdala MRI volume [55]. It is also well established that Aß oligomer species are neurotoxic and cause synaptic dysfunction [56, 57]. A recent study indicated that the earliest accumulation of Aß oligomers reduces the resting state connectivity of the precuneus [58].

The main advantages of higher field strength for functional MRI (fMRI) are the increased nuclear magnetization and susceptibility effects, leading to increased blood oxygenation level dependent (BOLD) contrast [59, 60] and therefore 7-T fMRI can provide up to 30 times higher spatial resolution than fMRI at 3 T and allow to gain new insights into brain dysfunction in AD. Of particular interest in AD is the dysfunction in hippocampal circuits. Currently, it is still unclear whether hippocampal dysfunction is related to the progression of tau- or amyloid-pathology or is related more to neurodegeneration (synaptic loss). This is an important question, because neurodegeneration-independent dysfunction is potentially reversible with treatments targeting tau- or amyloid-pathology. According to this possibility, circuit-specific dysfunction is the first impact of tau- and/or amyloid-pathology which is then followed by neurodegeneration. Currently, due to the limited resolution of 3 T, this question cannot be advanced much beyond what is already known. 7-T imaging, by enabling more sensitive measures of atrophy and brain function, can help to gain new insights into the question how neurodegeneration and/or synaptic dysfunction contribute to cognitive deficits and clinical disease progression.

Amyloid pathology can also be associated with intrinsic neuronal hyperexcitability of pyramidal neurons, which is already detectable at pre-plaque stages [61]. It is paralleled by inhibitory dysfunction which is thought to underlie the generation of network hyperexcitability and hypersynchrony that is observed in neurocognitive circuits of patients and of disease models [62]. Although fMRI studies are compatible with the presence of hyperactivity in preclinical and prodromal AD, thus far it could not be established whether increases in hippocampal activity reflect intrinsic hyperactivity or are rather a compensatory upregulation of activity in some subfields. 7 T based assessment of subfield-connectivity profiles could provide new insights into these questions.

The combination of ultra-high-resolution structural and functional imaging may be particularly powerful by allowing to assess the function of local circuits that are affected early by neurodegeneration and by allowing to quantify neurodegeneration precisely (Fig. 1). In the preclinical course of Alzheimer’s disease, tau-pathology spreads from perirhinal and entorhinal subregions to hippocampal subfields and amygdala and later to lateral temporal, frontal, and midline parietal regions [22, 63]. Therefore, 7T-based tools to assess the detailed functional connectivity profile of the hippocampus, its subfields and of the perirhinal and subregions of the entorhinal cortices in preclinical AD are expected to be highly valuable.

### Iron mapping

Iron dysregulation is thought to play a significant role in the pathogenesis of neurodegenerative diseases such as Alzheimer’s disease [64], Parkinson’s disease [65], and amyotrophic lateral sclerosis [66]. Large numbers of iron-laden glial cells are commonly found in the vicinity of pathological aggregates in these disorders [67–70]. QSM [71] and apparent transverse relaxation rate (R2*)—both related to brain iron levels in vivo—revealed differential patterns of involvement in aging [72–76] and Alzheimer’s disease [77–79]. QSM studies at 7 T may provide new insights into the role of iron-deposition in the pathophysiology of AD. In addition, QSM can help imaging venous vessels and their role in clearance of interstitial fluid and toxic waste and thus provide complementary information to TOF imaging of the arterial system. Finally, microbleeds can be well imaged with susceptibility-weighted imaging (see above) and imaging them may play an important role in the stratification for and monitoring of amyloid-modifying treatments where edema and microbleeds are major complications [80–84].

## Parkinson disease

Parkinson disease (PD) is a disabling neurodegenerative disorder, characterized clinically by motor and non-motor symptoms, and pathologically by synuclein intracellular inclusions with Lewy bodies formation.

Disease progression is characterized by the loss of dopaminergic neurons, the decreasing of neuromelanin content and the accumulation of iron in the substantia nigra (SN). The SN has been studied as the target of PD pathology with MR imaging since several decades; however, conventional MR sequences [85], segmented inversion-recovery ratio imaging [86] as well as DTI [87–89] did not enable the definition of the normal anatomy of the SN and have a limited role in diagnosing PD in individual subjects [90, 91].

Ultra-high-field (UHF) MRI offers several advantages for studying brainstem nuclei by enabling the assessment of their neuroanatomy and neurophysiology [92]. In particular, since magnetic susceptibility effects and signal phase tissue contrast increase with the static magnetic field (B0), higher contrast-to-noise ratio is achieved in susceptibility-weighted imaging at UHF. Moreover, the increase in signal-to-noise ratio (SNR) [93] can be spent to improve the spatial resolution in small brain structures such as the midbrain [92, 94]. With these premises, the introduction of 7-T MR equipment gave a new impulse to the imaging investigation of patients affected by PD: until the advent of 7-T MR, the evaluation of the substantia nigra, its inner structure, and its pathological changes in PD remained a prerogative of nuclear medicine, and neuroradiologic techniques were limited to the differential diagnosis.

### Iron sensitive 7-T MR of the substantia nigra

7-T MR has been used in PD to identify a radiological surrogate marker of nigral pathology to increase the diagnostic accuracy with respect to conventional MR systems.

**Fig. 2:**
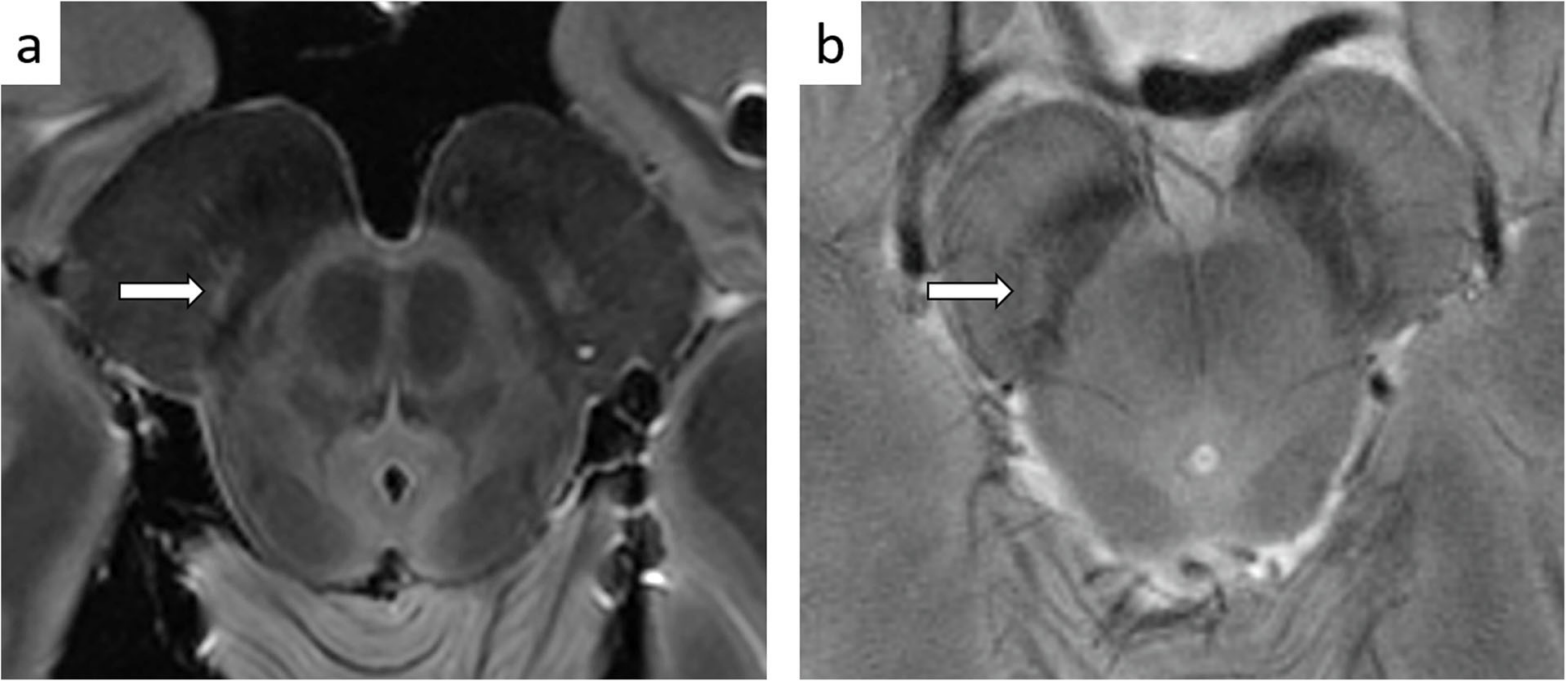
*Ex vivo* and *in vivo* MR imaging of the SN. The oval-shaped hyperintense formation indicated by arrows corresponds to nigrosome 1 (N1). **a** Proton density axial image of an *ex-vivo* sample. **b** Gradient echo imaging at high resolution allows to define the N1 formation with typical “swallow tail appearance” in healthy subjects *in vivo*

7-T MR imaging of the midbrain *ex vivo* and *in vivo* allows to depict the borders of the SN and its inner organization [95–97]. Iron-sensitive imaging sequences at 7 T targeting the midbrain demonstrated that the SN is structured into three tiers of signal intensity along the dorsoventral axis. From back to front, susceptibility-weighted images of the SN exhibit a thin hypointense signal band, followed by a high signal structure, which appears oval at the upper level, and, more anteriorly, by a large band of signal hypointensity extending until the crus cerebri (Fig. 2). The hyperintense ovoid area in the dorsolateral area of SN has been demonstrated to correspond to the largest nigrosome (nigrosome 1) [96] pertaining to calbindin-negative structures containing the neuromelanin of dopaminergic neurons and a low level of iron [98]. The nigrosome 1 has been variably described at 7 T [95, 97, 99] and finally the normal appearance of SN has been summarized with the term “swallow tail sign” [100] also at 7 T [101]. In PD, the nigrosome 1 is the most severely affected region of the SN [102]. The loss of signal hyperintensity of nigrosome 1 in PD is age-independent [103] and related to the loss of melanized neurons and to the increase of iron deposition [104] that, enhancing the magnetic susceptibility phenomena, masks the nigrosomal compartmental pattern based on calbindin of the SN [105]. The nigrosome 1 identification in healthy subjects [101] and its disappearance in PD has an outstanding diagnostic accuracy (sensitivity and specificity are respectively 100%, 92.3–100% [95]). Although MR signs of nigrosome 1 degeneration have been identified also at 3 T [100, 106] and are accepted in the clinical practice for diagnosing PD [107], in comparative studies the diagnostic accuracy at 3 T was about 10% lower than that at 7 T [108].

Ferric iron rich (paramagnetic) brain tissues can be conveniently studied with susceptibility-weighted imaging (SWI), an MRI technique that uses the information of both signal magnitude and phase. The information embedded in phase data can also be used to generate quantitative maps of magnetic susceptibility (QSM), which enable the measurement of local susceptibility and reflect the amount of iron content in the SN of PD patients [2]. An increase of QSM values in the SN of PD patients at 7 T has been reported [109], which has opened the perspective to quantify nigrosome degeneration along the disease course and its changes in response to therapy.

The immunohistochemical evaluation of the SN pars compacta reveals five calbindin-negative nigrosomes (N1 to N5) [98] that can be identified in *ex vivo* samples by using UHF MR [105]. All nigrosomes 1–5 have recently been detected *in vivo* in PD patients and controls using high-resolution iron-sensitive 7-T MRI. In PD patients the nigrosome 1 showed the most pronounced decrease in T2*-weighted signal and the best correlation to clinical scales (Unified Parkinson's Disease Rating Scale, UPDRS) even in the earliest stages of disease, confirming its role as a measure of disease severity [110, 111].

**Fig. 3:**
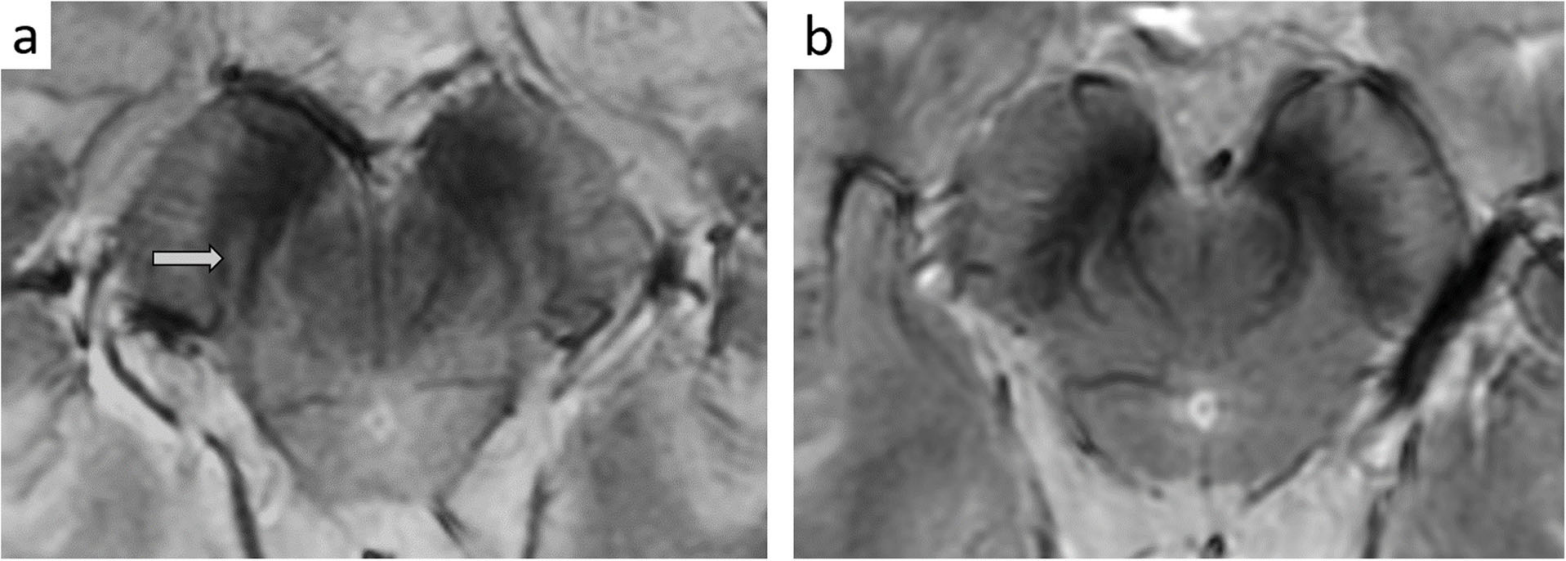
3D multi-echo T2*-weighted images of the substantia nigra at the level of the nigrosome 1 (arrow) in **a** an RBD patient with a normal imaging of the substantia nigra who has not developed symptoms or signs of parkinsonism in the follow-up; **b** an RBD patient with abnormal findings at imaging (the nigrosome 1 was not visible), who eventually converted to PD

Considering the long premotor period before the manifestation of motor symptoms, a preclinical diagnosis of PD would be desirable to test possible disease-modifying therapies. With this aim, a 7-T MR investigation of the midbrain has been attempted also in patients with predisposing conditions to develop PD. Carriers of gene mutation (parkin, PINK1, LRRK-2, DJ-1) [112], or patients with rapid eye movement behavior disorder (RBD) [113, 114] exhibited altered nigral anatomy with absent nigrosome representation in a preclinical condition (Fig. 3). Longitudinal studies on these subjects could provide important insights on the role of iron-sensitive 7-T MRI as a potential prognostic biomarker of neurodegeneration.

One of the main goals of MRI in PD is differential diagnosis. To investigate the causative role of nigrosome degeneration in PD, some studies have been conducted in patients with secondary parkinsonism without conventional MR abnormalities, such as drug-induced parkinsonism, and in patients with suspected vascular pseudoparkinsonism. Drug-induced parkinsonism occurs in the absence of presynaptic dopaminergic deficits: in such case, in accordance with the normal DAT scan, the visualization of nigrosome 1 is preserved [115]. Similarly, the nigrosome preservation characterizes patients with essential tremor, allowing a differential diagnosis with PD with high diagnostic accuracy [116]. These studies were conducted at 3 T and it can be expected that a comparative investigation with a 7-T system might reveal an increased confidence in the differential diagnosis.

The most frequent and challenging differential diagnosis attempted with 7-T MRI and iron sensitive sequences is between idiopathic PD and atypical parkinsonisms, particularly in the early stages of disease [117] when a significant number of patients have an incorrect clinical diagnosis.

UHF MR imaging studies of atypical parkinsonisms reported that anatomical changes of SN are not exclusive of PD; however, the impairment of the SN is not univocal in the different types of neurodegenerative parkinsonisms. The majority of patients with multiple system atrophy—clinical phenotype p (MSA-p) studied at 7 T have a loss of the signal hyperintensity corresponding to nigrosome 1, a pathological finding that is not invariably present in patients with MSA-c [99, 118]. A preservation of dopaminergic nigro-striatal function [119] reported with SPECT corresponds to an unremarkable imaging of the substantia nigra in some cases of corticobasal degeneration [120]. On the other hand, in progressive supranuclear palsy (PSP) the SN is invariably altered, with the loss of nigrosome 1 hyperintensity. In this type of atypical parkinsonism, the signal hypointensity at 7 T is more evident in the medial part of the pars compacta [120] where the iron deposition is prominent [121].

In summary, nigrosome imaging differentiates neurodegenerative from non-neurodegenerative parkinsonisms but, similarly to DATscan, is less effective in distinguishing PD from atypical parkinsonisms [122].

### Other potentialities for 7-T MR in PD

The dopaminergic neurons of the SN and the noradrenaline-containing neurons of the locus coeruleus (LC) contain high levels of neuromelanin, which is the target of neuromelanin-sensitive MRI. In PD, non-dopaminergic pathways such as noradrenaline neurons of the pons are involved in the neurodegenerative process [123].

The LC is the largest nucleus of noradrenergic neurons in the brain: it was indicated as the most affected extra-striatal area in PD [124], and neuromelanin depletion in the LC probably precedes that in the SN [125]. The depiction of the LC with MRI *in vivo* is based on the presence of neuromelanin, a paramagnetic pigment produced in noradrenergic neurons, and is achieved by using a T1-weighted Turbo Spin Echo sequence [126]. By leveraging on the different tissue relaxation times, which are field-strength-dependent, and on the increased SNR, which enables higher spatial resolution, several 7-T MR techniques provide detectable contrast between the LC and surrounding tissue. In particular, T1-weighted imaging with spectral presaturation inversion recovery (SPIR) provides higher contrast than Turbo Spin Echo (TSE)-based sequences at lower field strength. Notably, the small isotropic voxels that can be obtained at 7 T are an important advantage when visualizing small structures such as the LC [127]. The LC of patients with PD is currently under investigation also with Magnetization Transfer (MT)-weighted imaging at 7 T [128].

7-T MR spectroscopy has been used to investigate brainstem nuclei aiming to reveal non-dopaminergic system impairment that cannot be disclosed at conventional magnetic field strength. UHF provides not only improved SNR for MRS techniques, but also increased spectral resolution and reduced chemical shift dispersion of peaks [129]. Single-voxel 7-T MRS enabled the detection of metabolites including GABA [130]: in PD, increased GABAergic activity in the pons has been supposed to cause a reduction of excitatory outflow of the noradrenergic tone of the LC to the neurons of the substantia nigra. MRS at UHF could therefore reveal the earliest changes of metabolite concentrations in the brainstem of PD patients [131]. Indeed, UHF MRS of the brainstem revealed GABA increase in the pons relative to putamen in PD [132], indicating an earlier pathological involvement of the brainstem before nigrostriatal affection, according to the caudo-rostral spreading of synucleinopathy [133].

7-T MRI has also been aimed at to facilitate the surgical therapy of PD. Deep Brain Stimulation (DBS) is a well-established surgical technique for treating PD, consisting of the placement of stimulating electrodes within the motor component of the subthalamic nucleus (STN) to inhibit parkinsonian symptoms. The targeting of the STN can be done with stereotactic atlases or directly with MRI. Recently, the direct targeting of the STN [134] has been demonstrated to be feasible [135]. In addition, 7-T MRI data have been used to parcellate the globus pallidus into motor, associative and limbic regions in individual subjects to improve the precision of electrode placement [136]. Recently, a machine learning method based on 7 T data enabled the accurate prediction of the STN shape and position on the clinical image for targeting the STN in DBS [137], opening new perspectives in functional neurosurgery.

In conclusion, UHF MR in PD is currently used to identify a surrogate marker of disease with the aim to overcome the intrinsic limitations of conventional magnetic field strength MR, and until now 7 T has provided a better understanding of the anatomy and pathology of different brain structures involved in the pathologic processes in parkinsonisms.

## Amyotrophic lateral sclerosis

Amyotrophic lateral sclerosis (ALS) is a progressive and clinically heterogeneous neurological disease affecting both upper and lower motor neurons [138, 139]. Up to about 50% of patients also show cognitive or behavioral disturbances, and frontotemporal dementia is diagnosed in about 25% of these cases [140]. The etiopathogenesis of the disease is not completely known; both genetic and environmental factors have a pivotal role [141] and neuroinflammation, oxidative stress and glutamate induced excitotoxicity have been investigated as possible pathogenetic mechanisms [142–144].

The typical features of the upper motor neuron pathology are the loss of pyramidal cells of Betz in the layer V of the primary motor cortex together with the axonal loss and gliosis in the corticospinal tract; the lower motor neuron pathology, instead, is reflected by loss of motor neurons in the motor nuclei of the brainstem and in the anterior horn of the spinal cord [145, 146]. Moreover, reactive microglia/macrophages were found to be abundant in the affected areas of the brain and the spinal cord [145].

Even though MRI is not currently recommended for the specific search of brain abnormalities reported in ALS patients [147], imaging of the central nervous system has gained interest in the past two decades mainly with the purpose of finding, non-invasively, accurate biomarkers of disease which could aid early diagnosis and provide surrogate endpoints in clinical trials [148, 149]. Indeed, MRI can reveal the consequences of pathological changes, and MRS can detect the associated metabolic abnormalities. The advent of UHF (7-T) MR has further increased the interest in this field. At 7 T, the higher signal-to-noise ratio, sensitivity to magnetic susceptibility effects, spatial resolution of images, and spectral resolution of metabolites have allowed and could further enable in the future a more detailed depiction of morphological, metabolic and functional abnormalities in the brain and spinal cord of ALS patients, improving the accuracy in detecting motor neuron pathology with both qualitative and quantitative techniques.

### Brain

#### T2*-weighted imaging and quantitative susceptibility mapping

7-T MRI has enabled the detailed depiction of the radiological anatomy of the primary motor cortex in normal conditions and its changes in ALS patients. Even though MRI cannot directly reveal pathology at the cellular level, it can show the consequences of Betz cells loss and intracortical accumulation of microglia. As neuronal loss can be revealed by the cortical thinning, the presence of ferritin-laden microglial cells can be unveiled indirectly by the abnormal hypointense rim in the motor cortex in T2*-weighted imaging. The high ferritin content in microglial cells, whose meaning in the disease process has not been fully clarified yet, causes the shortening of the relaxation time T2 and the consequent T2* hypointensity [69].

**Fig. 4:**
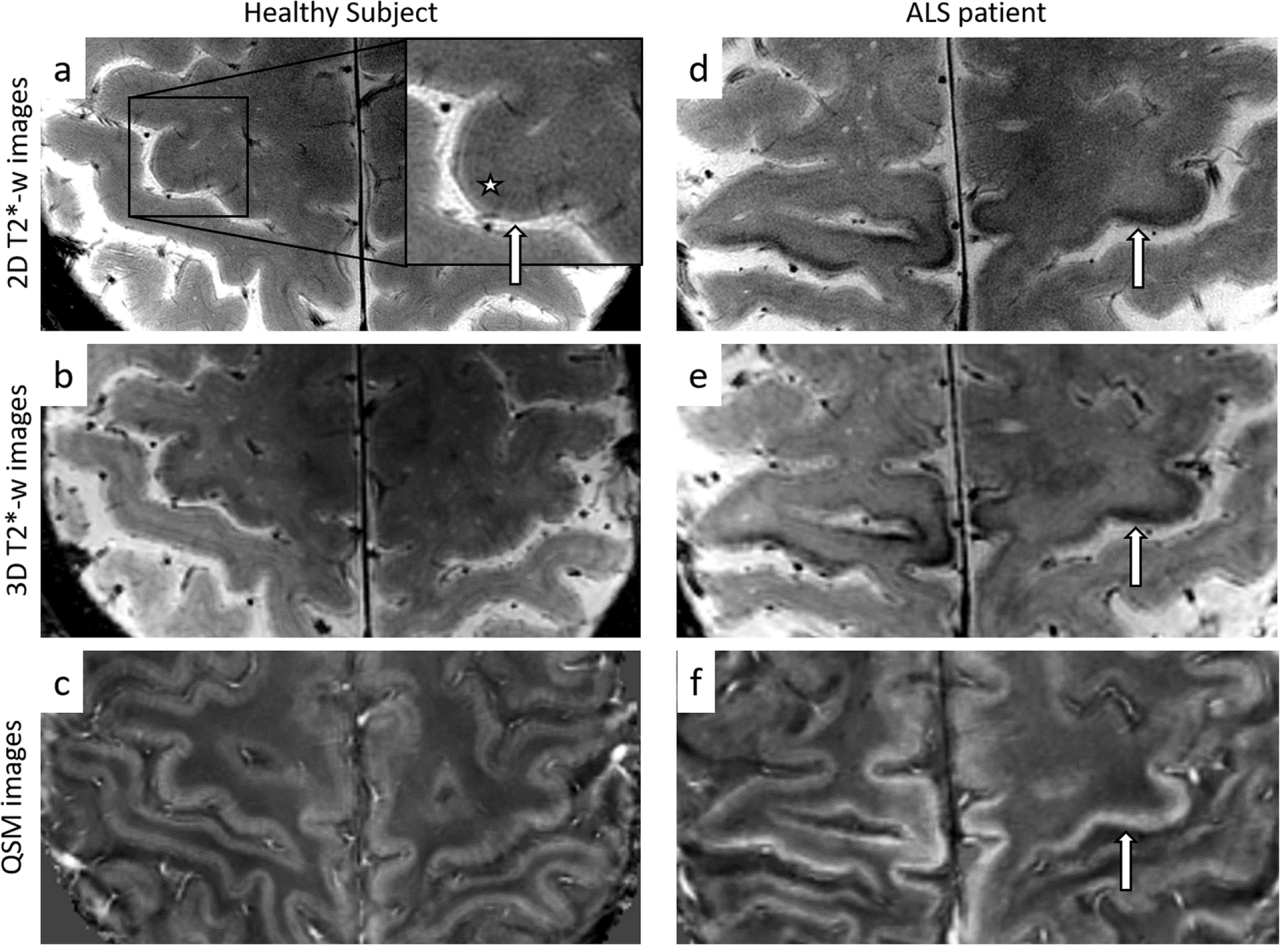
T2*-weighted images and QSM images of the primary motor cortex in a healthy subject (**a**–**c**) and an ALS patient (**d**–**f**). **a** In healthy subjects, two strips are recognisable in the primary motor cortex: a thin superficial hyperintense strip (arrow) and a thicker and slightly hypointense deep band (*). In many ALS patients, the deep strip is abnormally hypointense in T2*-weighted images (arrows in **d** and **e**), and the hypointensity corresponds to higher values of magnetic susceptibility (arrow in **f**)

The primary motor cortex was shown to have a typical MR appearance characterized by a thin superficial hyperintense strip, always preserved in ALS patients, which lies just above a thicker and slightly hypointense band, radiologically affected in ALS [150] (Fig. 4). In many patients, in fact, the deep strip was shown to be thinner and more hypointense than normal [150] (Fig. 4d, e), with fewer pyramidal cells of Betz and many ferritin-laden microglial cells [69].

The cortical signal hypointensity, described for the first time in T2-weighted images of ALS patients in 1993 [151, 152] and reported with variable sensitivity [153, 154], was proved to be more visible and accurate in identifying patients at 7 T than at 3 T [155, 156]. It has been proposed as a sign of upper motor neuron impairment [152] and that hypothesis has been confirmed in recent studies. The regional grade of cortical atrophy and hypointensity was found to correspond to a somatotopic functional disability related to the upper motor neuron pathology [150] and can differ from one body region to another [150, 155]: the lower the T2* signal intensity and the thickness of the deep layers of the primary motor cortex, the worse the upper motor neuron impairment of the corresponding body regions [150].

The signal hypointensity has been further investigated and the iron concentration estimated using QSM. Differently from conventional T2*-weighted sequences, quantitative susceptibility maps provide objective and more reproducible data for cortical assessment. Even though the mean magnetic susceptibility of a tissue is influenced by all the components, iron is the prevalent source of magnetic susceptibility-based contrast in the cerebral cortex [157] and its expected concentration has been proven to correlate with magnetic susceptibility measures [158]. The marked hypointensity in the primary motor cortex of ALS patients was confirmed to be related to paramagnetic tissue [158] (Fig. 4f); moreover, the cortical magnetic susceptibility, which is associated with the degree of microglial activation [159], was found to significantly correlate with the clinical upper motor neuron impairment [158].

Therefore, although cortical atrophy, hypointensity, and increased magnetic susceptibility were known findings in many ALS patients also at conventional magnetic fields [151–153, 155, 160–163], their characterization and accuracy in diagnosing the upper motor neuron pathology have been improved by using 7-T MR systems.

#### Magnetic resonance spectroscopy

MRS can reveal metabolic changes of the brain related to one or more key points of ALS pathology. This is true at conventional magnetic fields and even more so at 7 T, where the higher signal-to-noise ratio and spectral resolution can lead to an increased precision in metabolite quantification and detectability of low concentration metabolites [164].

Both ^1^H and ^31^P MRS have been employed at 7 T: the former has enabled the assessment of changes in the tissue concentration of mainly N-acetylaspartate (NAA, a marker of neuronal density and integrity [165]), myo-inositol (mI, a marker of glial cells [166]), glutamate (Glu, the main excitatory neurotransmitter), and gamma-aminobutyric acid (GABA, the main inhibitory neurotransmitter in the cortex); ^31^P 7-T MRS has been employed to investigate the energetic status of the cells and the membrane metabolism.

##### MRS in ALS patients

There is only a small number of published studies investigating metabolic changes in ALS patients at 7 T and they focused mainly on the primary motor cortex [167, 168]. Both NAA and total NAA (tNAA) were found to be significantly decreased in the precentral gyrus of ALS patients compared to controls [167], and the level of tNAA was shown to depend on the diagnostic subcategory, with probable/definite ALS being more affected than possible ALS [168]. Compared to controls, mI was higher in the primary motor cortex of patients, in particular in probable/definite ALS patients [168].

Reported results about Glu are conflicting, with significantly reduced levels in the precentral gyrus of patients in one study [167] but not in the other [168]. Interestingly, the significant and positive correlation between Glu and NAA levels suggests that the Glu reduction in ALS patients is driven by neuronal loss [167]. On the contrary, GABA was not found to be significantly different between patients and controls [167].

The effect of taking riluzole, a glutamatergic neurotransmission inhibitor [169], was also investigated. Addressing the suggested glutamate induced excitotoxicity pathogenetic mechanisms of ALS, riluzole might influence levels of Glu but also those of metabolites related to neuronal density and neuroinflammation. Even though no difference was observed between riluzole-treated and riluzole-naive patients, riluzole-naive patients showed lower tNAA/mI than controls [168].

Another topic in MRS is the search for a relationship between clinical data and metabolite concentrations or ratios. At 7 T, the greater disease severity (assessed with the Revised Amyotrophic Lateral Sclerosis Functional Rating Scale, ALSFRS-R) was found to be associated with lower levels of tNAA, tNAA/tCr, tNAA/mI, and Glu in the precentral gyrus [168], and the heavier clinical upper motor neuron impairment with higher mI/tNAA in the motor cortex only in one study [167]. Unfortunately, different research groups employ different clinical scales to assess upper motor neuron dysfunction, and they could have different sensitivity to pathology.

When interpreting results of MRS studies, some factors have to be considered. In single voxel studies, a limited part of the potentially affected tissue is investigated, and disease duration at the time of the MR acquisition and clinical phenotype can influence the results. Synaptic concentrations of Glu and GABA might be influenced by riluzole [169, 170], the precision of GABA quantification might not to be sufficient even at 7 T [168], and Glu is located in both the intracellular and extracellular space, even though the extracellular concentration is much higher than the intracellular one [171]. Therefore, changes in Glu concentration revealed by MRS could be driven by the reduced intracellular concentration via neuronal loss, increased extracellular concentration responsible for excitotoxicity or both, with the possible influence of riluzole.

##### MRS in asymptomatic C9orf72 repeat expansion carriers

C9orf72 hexanucleotide repeat expansion is the most frequent gene mutation in both familial and sporadic ALS cases [172, 173] and is associated with an almost full penetrance by 80 years [172]. Therefore, investigating asymptomatic C9orf72 repeat expansion carriers might provide non-invasively new insights into the pathophysiology of ALS and possible pre-symptomatic alterations, and some metabolic changes have been documented in many brain regions using both 1H MRS and 31P MRS.

Compared to non-carriers, in C9orf72 repeat expansion carriers lower levels of tNAA/tCr and Glu/tCr were found in the left putamen and lower levels of Glu/tNAA were observed in the putamen and thalamus of the left hemisphere [174]. These results might reflect a lower concentration of neurons and Glu per neuron, whose meaning needs further research to be elucidated. Moreover, glycerophosphoethalonamine-to-phosphocreatine ratio (GPE/PCr) and uridine diphosphoglucose-to-phosphocreatine ratio (UDPG/PCr) were found to be significantly higher in many brain regions of asymptomatic carriers [175]. Being GPE one of the cell membrane degradation products and UDPG a precursor of glycogen, these results might reflect an increased catabolism of the cell membranes and an imbalance of energy metabolism, respectively [175].

In conclusion, MRS studies at ultra-high-field confirmed lower levels of NAA and higher levels of mI in the precentral gyrus of ALS patients, provided new insight into the pre-symptomatic brain changes, but showed inconsistent results about Glu. Further studies including larger cohorts of patients and subjects at high risk of developing the disease are needed, using whole-brain and multi-nuclear MRS, to explore metabolic brain changes and possible patterns related to diagnostic subcategories, clinical phenotypes or pharmacological treatments.

#### Quantitative T1 mapping, magnetization transfer contrast, and amide proton transfer-weighted imaging

The corticospinal tract has been widely investigated in ALS using DTI at conventional magnetic fields, and reduced fractional anisotropy was consistently reported [176]. The basis for this diffusivity change, explored at 7 T by combining quantitative T1 mapping, magnetization transfer ratio and amide proton transfer-weighted imaging, might be more likely related to gliosis and expansion of the extracellular matrix rather than to demyelination [177].

### Spinal cord

While UHF MR has the potential to greatly improve spinal cord imaging, several technical issues, such as physiological noise and inhomogeneities of the static magnetic and radiofrequency fields, have limited its application in clinical studies. A number of possible solutions have been and are being explored to overcome these limitations [178]. Recent studies have shown the feasibility of conventional imaging [179], MRS [180], glutamate-weighted chemical exchange saturation transfer (CEST) MR imaging [181] and diffusion tensor imaging of the spinal cord [182], opening the door to their possible future applications in clinical research. So far, only two studies investigating the spinal cord in ALS patients at 7 T have been published; one study assessed *in vivo* alterations [183] and the other made a comparison between MRI and histology in an *ex vivo* specimen [159].

#### Conventional imaging

Imaging the spinal cord at very high resolution (about 200–400 μm in-plane) can enable the depiction of alterations occurring in ALS patients. It has the potentiality of representing in the same images signal changes of the lateral columns and the atrophy of the anterior horn of the spinal cord, which reflect upper and lower motor neuron degeneration, respectively.

Signal hyperintensity in the lateral segments of the cervical spinal cord has been documented at 7 T in one ALS patient [183] and in an *ex vivo* specimen [159] but not in a control subject [183]. The location of such signal alteration in patients together with the presence of histological signs of degeneration [159] have suggested the hyperintensity as a sign of corticospinal tract degeneration [183].

On the other hand, the detailed differentiation between white and grey matter and the measurement of the grey matter area have been proven to be feasible at 7 T [179] but not employed yet in clinical studies.

#### Diffusion tensor imaging

So far, there are no published accounts in the literature about DTI of the spinal cord at 7 T in ALS patients in vivo. However, in an ex vivo specimen, the signal hyperintensity and histological fibre degeneration in the lateral segments of the cervical spinal cord were found to parallel the significant decrease of fractional anisotropy and increase of mean diffusivity [159]. DTI metrics can therefore contribute to further assess the upper motor neuron pathology in the spinal cord of ALS patients.

In conclusion, magnetic resonance techniques at 7 T have provided new insights into the pathophysiology of ALS. They have allowed revealing morphological, quantitative and metabolic changes in the central nervous system, mainly in the primary motor area and in the corticospinal tract, which are related to pathologic abnormalities that occur in the disease. Technical developments and overcoming current issues and limitations will open interesting prospects for future research.

## Conclusion

This review has described how UHF MR has recently impacted on clinical studies addressing AD, PD, and ALS and provides new avenues for research. Researchers have capitalized on higher SNR, signal sensitivity, and improved spatial resolution offered by UHF-MRI, to obtain a clearer depiction of the anatomical regions involved in each disease. The pathological changes of brain function and circuitry have been studied with fMRI with improved BOLD contrast and spatial detail, while the increased spectral resolution and reduced chemical shift dispersion enabled by UHF MRS have shed new light on the metabolic changes occurring in each pathology.

The introduction of UHF scanners allowed to identify radiological signs in neurodegenerative disorders, previously undetectable at magnetic fields ≤ 3 T: the detection of structural and functional degeneration within the hippocampus of AD patients, loss of anatomical integrity of the substantia nigra in PD and increased magnetic susceptibility in the primary motor cortex of ALS patients with upper motor neuron degeneration are examples of the clinical/radiological impact deriving from the current research in neurodegenerative disorders with UHF.

UHF scanners have also offered new ground for more advanced and challenging techniques, such as CEST imaging and magnetic resonance of nuclei other than ^1^H (x-nuclei). However, at the present time, the use of such techniques is still little documented in the published literature on neurodegenerative diseases.

While on the one hand, there is no doubt about the clear advantages offered by UHF MR, and on the other hand, some limitations still restrict its applicability mostly to the field of clinical research. However, the steady technological progress in MR hardware and acquisition techniques, including for example the development of improved parallel imaging transmission systems, gradient coils, and prospective motion correction methods, together with the recent introduction in the market of 7 T scanners certified for diagnostic use, suggest that in the near future UHF MR might have a further increasing impact in the study and diagnosis of AD, PD, and ALS.

## Data Availability

Not applicable (literature review).

## Abbreviations

AD: Alzheimer’s disease
ALS: Amyotrophic lateral sclerosis
CSF: Cerebro spinal fluid
DBS: Deep brain stimulation
DTI: Diffusion tensor imaging
LC: Locus coeruleus
MR: Magnetic resonance
MRI: Magnetic resonance imaging
MRS: Magnetic resonance spectroscopy
MSA: Multiple system atrophy
PD: Parkinson’s disease
QSM: Quantitative susceptibility mapping
RBD: Rapid-eye-movement behavioral disorder
SN: Substantia nigra
SNR: Signal-to-noise ratio
STN: Sub thalamic nucleus
SWI: Susceptibility-weighted imaging
TOF: Time of flight
UHF: Ultra-high field
